# Progressive Familial Intrahepatic Cholestasis: A Descriptive Study in a Tertiary Care Center

**DOI:** 10.1155/2023/1960152

**Published:** 2023-07-20

**Authors:** Fahad I. Alsohaibani, Musthafa C. Peedikayil, Abdulaziz F. Alfadley, Mohamed K. Aboueissa, Faisal A. Abaalkhail, Saleh A. Alqahtani

**Affiliations:** ^1^Department of Medicine, King Faisal Specialist Hospital and Research Center, Riyadh, Saudi Arabia; ^2^College of Medicine, Alfaisal University, Riyadh, Saudi Arabia; ^3^Liver Transplant Centre, King Faisal Specialist Hospital and Research Centre, Riyadh, Saudi Arabia; ^4^Division of Gastroenterology and Hepatology, Johns Hopkins University, Baltimore, Maryland, USA

## Abstract

**Background:**

Progressive familial intrahepatic cholestasis (PFIC) is a rare genetic disorder that results from defective mechanisms of bile secretion. We aim to describe different types of PFIC and their clinical features, treatment modalities, and outcomes in Saudi Arabia. *Patients and Methods*. This is a retrospective study of all patients diagnosed with PFIC at King Faisal Specialist Hospital and Research Center in Riyadh from January 1, 2002, to December 31, 2021. All relevant information was collected from patient charts and transferred into the REDcap® database for statistical analysis.

**Results:**

A total of 79 patients were identified with PFIC, and PFIC type 3 was the most common (59.5%), followed by PFIC type 2 (34.2%), PFIC type 1 (5.1%), and PFIC type 4 (1.3%). Males and females were affected in 54.4% and 45.6%, respectively. Mutations in ATP8B1, ABCB11, and ABCB4 genes were observed in PFIC type 1, PFIC type 2, and PFIC type 3, and loss of function in a variant of TJP2 was detected in PFIC type 4, respectively. A total of 51 (64.6%) patients underwent liver transplantation: three patients (3/4) with PFIC type 1 (75%), twenty patients (20/27) with PFIC type 2 (74.1%), twenty-seven patients (27/47) with PFIC type 3 (57.4%), and one patient with PFIC type 4 (100%). The mean duration of disease before transplantation was 53.9 ± 67 months with a median of 30 months. Following liver transplantation, symptomatic control was achieved in 47 patients (92.2%). Recurrence after transplantation occurred in 4 patients (7.8%) within an average of 22.5 months and a median of 17 months.

**Conclusion:**

PFIC is considered a rare disorder in Saudi Arabia; however, early recognition of the disease is important for appropriate management and early referral for liver transplantation evaluation. The overall rate of liver transplantation in our cohort was 64.6% with an excellent five-year survival rate.

## 1. Background

Progressive familial intrahepatic cholestasis (PFIC) is a rare group of autosomal recessive inherited diseases characterized by intrahepatic cholestasis and manifests with jaundice, pruritus, and failure to thrive in infants and children and usually advances to end-stage liver disease [1–3]. It accounts for 10-15% of neonatal cholestasis syndrome, and 10-15% of children who require liver transplantation are attributed to PFIC [2, 4]. Based on genetic mutations and clinical manifestations, PFIC is now divided into six subtypes (I to VI). PFIC was previously divided into three types based on the mutated genes of ATP8B1, ABCB11, and ABCB4; however, with the development of diagnostic methods, such as next-generation sequencing and whole-exome sequencing, new mutated genes have been detected in recent years, such as TJP2, NR1H4, and MYO5B, that are responsible for PFIC types IV, V, and VI, respectively [5, 6]. Worldwide, PFIC has an estimated incidence of 1 per 50,000-100,000; however, the exact incidence of PFIC in Saudi Arabia is unknown. It is known that clinical presentation and laboratory findings in patients with PFIC can overlap with other cholestatic liver diseases [7, 8]. In this study, we aimed to explore PFIC in Saudi Arabia which included clinical manifestations, diagnostic methods such as genetics and histology, medical and surgical treatment including liver transplantation, and their outcome.

## 2. Patients and Methods

This is a retrospective study conducted at King Faisal Specialist Hospital and Research Center, Riyadh, Saudi Arabia. The patients were selected from January 1, 2002, to December 31, 2021. The patients with a diagnosis of PFIC that was based on genetic tests with specific culprit mutations were included. The data were collected and entered using the REDcap® application for statistical analysis. All information related to patients' demographics, clinical presentation, laboratory results, liver histopathology, genetic analysis, and treatment modalities was collected. The time from clinical presentation to the diagnosis was documented, and if the disease was initially misdiagnosed with another hepatic disease, the overall prognosis and outcome post liver transplantation including recurrence of the disease were also collected and reported.

### 2.1. Statistical Analysis

Statistical analysis was done using JAMOVI. Descriptive statistics were used to summarize the continuous variables. The categorical variables were expressed as proportions, while the continuous variables were expressed as medians and/or means. Pearson's chi-square test was used to compare categorical variables and the *t*-test for comparing continuous variables. A two-tailed *P* value of < 0.05 was considered statistically significant.

### 2.2. Ethical Considerations

All the required data in this study already exist in the medical records and were collected after a waiver of consent was obtained. The research proposal was reviewed and approved by the Institutional Review Board (IRB) with Research Advisory Council (RAC #2191180).

## 3. Results

**Figure 1:**
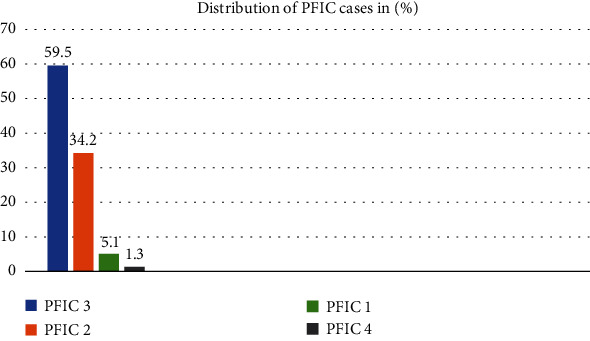
Frequencies of PFIC patients (%).

We identified 79 Saudi patients with PFIC by searching electronic medical records. The frequency of different types of PFIC is shown in Figure 1 with PFIC type 3 being the most common (59.5%) followed by PFIC type 2 (34.2%), PFIC type 1 (5.1%), and PFIC type 4 (1.3%).

Males were 54.4% with a mean age of 68.3 months ± 66.2 and a median of 37 months (Min: 3–Max: 293). Females were 45.6% with a mean age of 56.8 months ± 53.1 and a median of 44 months (Min: 1–Max: 188). The overall mean age at diagnosis was 63.1 ± 60.5 months with a median of 35.5 months (Min: 1–Max: 239). The regional distribution of the patients was as follows: southern region (30%), central region (28%), western region (24%), eastern region (14%), and northern region (4%).

Data on consanguineous marriage was available from 54 patients and 87% of them; their parents were married to second-degree relatives. A positive family history of PFIC was observed in 61.8% of cases. Jaundice, pruritus, and failure to thrive at presentation were seen in 49.4%, 48.1%, and 10.1%, respectively. Splenomegaly was seen in 65.8% and hepatomegaly in 62% of patients. None of the patients had sensorineural deafness or a history of pancreatitis. Fifteen patients (18.9%) were initially diagnosed with different diseases (7 patients as Wilson's disease, 3 patients as primary sclerosing cholangitis, and 1 patient each as Alagille's syndrome, autoimmune hepatitis, atopic dermatitis, autoimmune cholangitis, and hypoplastic biliary tree). Overall baseline characteristics are given in Table 1.

### 3.1. PFIC Type 1

Two males and two females were diagnosed with PFIC type 1. Their mean age was 9.75 years. For the two females, none of the family members were affected. The first female patient presented at the age of 3 months with hepatosplenomegaly, jaundice, and diarrhea. She was diagnosed 24 months after the presentation and underwent liver transplantation. The second female patient had decompensated liver disease and hepatomegaly at presentation and underwent a liver transplant two months later. The first male patient presented with jaundice, pruritus, and hepatosplenomegaly at the age of 1 month and underwent liver transplantation at the age of 9 months. He had 3 siblings: 2 sisters and 1 brother who died at the age of 2 years because of an unknown liver disease. The second male patient was referred to our hospital at the age of 9 months; his symptoms started at the age of 2 weeks with jaundice, and then, pruritus started at the age of 3 months. He had a history of diarrhea and failure to thrive since birth. He underwent liver transplantation at the age of 2 years.

### 3.2. PFIC Types 2 and 3

The gender distribution was equal for both types. Forty percent of the patients with PFIC type 3 came from the south, and 33% of PFIC type 2 came from the central and western areas; however, this was not statistically significant. Consanguinity between parents was observed in 89.5% and 86% for PFIC type 2 and PFIC type 3, respectively. Similarly, there were no differences in the symptoms, physical signs, and extrahepatic manifestations of patients with PFIC types 2 and 3. The mean and median values of bile acids, bilirubin, alanine aminotransferase, and alpha-fetoprotein were significantly high in patients with PFIC type 2. Gamma-glutamyl transferase (GGT) was normal in PFIC type 2 and significantly higher in PFIC type 3. Patients with PFIC type 2 were diagnosed at a mean age of 27.36 months compared to 83.4 months in PFIC type 3 (*P* value 0.01). Differences in baseline characteristics, clinical manifestations, and laboratory investigations of PFIC type 2 and PFIC 3 are displayed in Tables 2 and 3.

### 3.3. PFIC Type 4

PFIC type 4 was diagnosed only in one male patient at the age of three years. He presented with jaundice and pruritus. He underwent liver transplantation at the age of four years due to end-stage liver disease.

### 3.4. Diagnosis

The results of genetic mutations were documented for all 79 patients. 37 patients' genetic mutations were identified. 31 patients were homozygous (PFIC type 1: one, PFIC type 2: ten, PFIC type 3: nineteen, and PFIC type 4: one), and six were heterozygous (PFIC type 1: two, PFIC type 2: two, and PFIC type 3: two). From our cohort of 79 PFIC patients, 53 of them (67.1%) underwent liver biopsy. The histopathological appearance of the liver biopsy was typical for PFIC in 39 patients (73.6%). Typical histological features of each type of PFIC are displayed in Table 4 [5].

### 3.5. Treatment and Outcome

#### 3.5.1. Medical Treatment

Ursodeoxycholic acid (UDCA) was used for the treatment of pruritus in 51 (64.6%) patients and rifampin in 14 (17.7%). Response to treatment by any symptomatic improvement and liver function tests was variable.

#### 3.5.2. Surgical Treatment

Only one patient (PFIC type 2) was treated with internal biliary drainage at age 2 years. The patient had a heterozygous mutation in ABCB11 (c.2944G>A). He continued to have significant itching despite three 3 antipruritic medications (cholestyramine, rifampin, and phenobarbital) and UDCA. At age five, she underwent orthotropic liver transplantation for decompensated liver disease, and she is currently on UDCA and tacrolimus. The patient is asymptomatic and doing well.

#### 3.5.3. Liver Transplantation (LT)

A total of 51 (64.6%) patients underwent liver transplantation (LT) (3/4 (75%) in PFIC type 1, 20/27 (74.1%) in PFIC type 2, 27/47 (57.4%) in PFIC type 3, and one in PFIC type 4 (100%)) (Table 5). The mean duration of the disease before transplantation was 53.9 ± 67 months. Following liver transplantation, symptomatic control was achieved in almost all patients (92.2%). Long-term immunosuppressive therapy in the form of tacrolimus was in 86% of patients; similarly, mycophenolate mofetil was used in 32%, corticosteroid in 32%, sirolimus in 8%, and everolimus only in one patient (2%). Details on liver transplantation are given in (Table 5).

#### 3.5.4. Recurrence after Liver Transplantation

Post liver transplantation, four patients (7.8%) developed recurrence of the primary disease within mean months of 22.5 ± 23 (median of 17 (Min: 1–Max: 55 months)). All four patients were PFIC type 2, and three of them had homozygous mutations. The recurrence of PFIC was treated with a protocol-based regime. The treatment of recurrence and their responses are given in (Table 6).

#### 3.5.5. Hepatocellular Carcinoma (HCC)

During follow-up, hepatocellular carcinoma (HCC) developed in four patients; two of them were PFIC type 2, and the other two were PFIC type 3. The first (PFIC type 2) patient developed multifocal HCC requiring a living donor liver transplantation in 2014. The patient is currently doing well and asymptomatic. The second (PFIC type 2) patient had a unifocal HCC requiring living donor liver transplantation in 2012. The patient is currently stable and clinically asymptomatic apart from mild intermittent pruritus. The third patient (PFIC type 3) had a multifocal HCC in 2011; for that, he had radiofrequency ablation and then underwent living donor transplantation a month later. In 2018, due to medication nonadherence, he developed graft rejection that responded well to corticosteroids, and currently, he is asymptomatic. The fourth (PFIC type 3) patient had a unifocal HCC requiring radiofrequency ablation and then cadaveric liver transplantation in 2017 but developed acute graft rejection requiring second cadaveric liver transplantation in the same year, and he is currently alive and well.

#### 3.5.6. Mortality

Three patients died during follow-up; all of them had PFIC type 3. One patient was listed for liver transplantation, but unfortunately, he passed away due to septic shock and multiorgan failure. Another patient underwent liver transplantation and passed away five days later due to multiorgan failure, brain edema, and herniation. The third patient died two weeks after liver transplantation because of acute rejection and upper gastrointestinal hemorrhage and refractory acidosis.

## 4. Discussion

PFIC is a rare genetic disorder that was first described by Clayton et al. in 1965 as Byler's disease in a population of Amish kindred [9]. PFIC type 1, also known as Byler's disease, is an autosomal recessive disease caused by homozygous or compound heterozygous mutations of the ATP8B1 (adenosine triphosphatase, type 8B, member 1, formerly named FIC1) gene on chromosome 18 locus q21-22, which encodes the FIC1 protein. FIC1 preserves the asymmetrical phospholipid distribution across the canicular membrane; hence, it protects the membrane from bile acids [5, 6]. PFIC type 2 is caused by a mutation in the ABCB11 (adenosine triphosphate-binding cassette, subfamily B, member 11) gene. This gene encodes bile salt export pump (BESP), a protein responsible for transporting bile salts against their concentration gradient [5, 6]. Therefore, a mutant BSEP leads to the accumulation of bile salts in hepatocytes, leading to hepatocellular damage [2]. BSEP deficiency can be subdivided into BSEP1 (p.D482G (c.1445A>G) or p.E297G (c.890A>G) mutation), BSEP2 (at least 1 missense mutation, not p.D482G or p.E297G), or BSEP3 (mutations leading to a predicted nonfunctional protein). Patients with the BSEP1 genotype have residual BSEP functionality compared to patients exhibiting BSEP2/BSEP3 genotype; hence, it presents with a milder phenotype compared to the other genotypes [5].

PFIC type 3 is due to a defective ABCB4 (adenosine triphosphate-binding cassette, subfamily B, member 4) gene, which encodes a multidrug-resistant class III glycoprotein (MDR3) [1]. The MDR3 protein functions as a phospholipid transporter across the hepatocyte canicular membrane [10]. Normally, bile salts are neutralized in the presence of phospholipids [11, 12]. However, in MDR3-affected patients, phospholipids are not transported to the bile canaliculi; hence, bile salts become insoluble. Therefore, stones and hepatocellular damage ensue [5]. PFIC type 4 is caused by a loss-of-function mutation in the tight junction protein 2 (TJP2), also called zona occludens 2, present on chromosome 9q21. PFIC type 5 results from mutations in the NR1H4 gene (chromosome 12q23), encoding FXR (farnesoid X receptor) gene [5, 6, 13–17].

In our study, we aimed to determine the distribution, disease characteristics, and clinical outcomes of patients with PFIC in Saudi Arabia. We found that the most common type of PFIC was PFIC type 3 (59.5%) followed by PFIC type 2 (34.2%), PFIC type 1 (5.1%), and PFIC type 4 (1.3%), compared to many reports from other countries that estimate that the majority of individuals with PFIC have PFIC type 1 or type 2, while PFIC type 3 is present in 33% [15, 18]. Although PFIC types are different, they share similar clinical presentation, mainly pruritus, and jaundice. However, PFIC type 1 and PFIC type 2 present early in life while PFIC type 3 presents in the second decade of life [1, 5, 11]. In our study, PFIC type 2 patients presented at an average of 27.36 months when compared to 83.4 months in PFIC type 3.

In biochemical studies, both PFIC types 1 and 2 patients share normal serum gamma-glutamyl transferase (GGT) activity, normal serum cholesterol levels, and high serum bile acid concentrations [1]. However, PFIC type 2 patients at diagnosis have higher transaminase and alpha-fetoprotein serum levels than PFIC type 1 patients [1, 19, 20]. This was also reflected in our study as patients with PFIC type 2 had significantly high bile acids, bilirubin, alanine aminotransferase, alkaline phosphatase, and alpha-fetoprotein when compared to PFIC type 3 patients. As expected, the mean serum GGT levels were significantly high in PFIC type 3. As expected, patients with PFIC type 3 have persistently high serum GGT activity, normal serum cholesterol levels, and elevated serum primary bile salt concentrations [1, 19, 20]. In our cohort, we were able to identify genetic mutations in 79 patients. The diagnosis of PFIC was often delayed or wrongly diagnosed as another form of liver disease. Fifteen (19%) of the patients in our study were initially diagnosed with another form of liver disease.

Patients present with abnormal liver function tests due to intrahepatic cholestasis. PFIC can lead to progressive chronic liver disease because of impaired bile flow through the liver caused by the mutant protein. Patients develop persistent jaundice at an early stage in life, growth retardation, malabsorption, and pruritus, and most cases of PFIC present in infancy or early childhood with jaundice and cholestasis progress rapidly to fibrosis and end-stage liver disease. If left untreated, end-stage liver disease will result in hepatic decompensation and mortality [1, 4, 21].

In our current study, four cases of HCC were diagnosed; two of them were in PFIC type 2, and the other two were in PFIC type 3 patients. Hepatocellular carcinoma HCC is well documented in PFIC type 2 patients [5, 14, 22]. In addition, HCC incidence increases with the genotype severity from 4% in BSEP1 to 7% in BSEP2 and 34% in BSEP3 [5]. However, HCC was not seen in patients with PFIC type 1 [1, 5, 14, 22]. In PFIC type 3 patients, approximately 20% of cases will develop HCC between the 2nd and 7th decade of life [4, 23].

The initial therapeutic management of children with all types of PFIC is UDCA [2, 5, 14, 24]. It is shown to be an effective treatment for pruritus in PFIC type 2 and can reverse liver fibrosis in PFIC type 3 [2, 5]. In our study, UDCA was used for the treatment of pruritus in 51 (64.6%) patients. Surgical procedures include biliary diversion, liver transplantation, and ileal exclusion. Surgical biliary diversion is considered a first-line treatment option as it slows down disease progression and may improve pruritus; however, once cirrhosis develops, biliary drainage is ineffective and linked with poor clinical outcomes [25]. In our study, only one patient was treated with partial internal biliary diversion. None of our patients underwent partial cutaneous biliary diversion or ileal bypass surgeries.

Liver transplantation is a proven curative treatment for PFIC-related end-stage liver disease [19, 20, 22, 26]. It is considered when patients have failed medical treatment, biliary diversion, or refractory pruritus. LT is also considered when patients have decompensated liver disease or HCC within Milan criteria [5, 14]. Data on liver transplantation for patients with different kinds of PFIC are increasing [5, 14]. However, experience with liver transplantation in PFIC type 3 patients compared to other types is limited. From our center, a total of 51 (64.6%) patients underwent LT; out of that, a total of 27 patients were PFIC type 3. Recurrence after transplant occurred in 4 (7.8%) patients. Two patients died after transplantation, and both patients were PFIC type 3. From our experience, if patients are appropriately selected, liver transplantation offers an excellent survival benefit. A review article also supports this as it found that liver transplantation in 117 patients with PFIC had graft survival ranges of 69.2% to 100% and patient survival range from 73% to 100% [14].

Odevixibat is a novel treatment for PFIC. It is a small molecule inhibitor of the ileal bile acid transporter. In 2021, it was approved in the United States for the treatment of pruritus in patients aged ≥3 months with PFIC and in the European Union for the treatment of PFIC in patients aged ≥6 months [27]. Inhibition of the bile acid transporter blocks the reabsorption of bile salts in the terminal ileum and thereby lowers levels of serum bile acids that are raised in patients with PFIC. It acts locally on the bile acid transporter in the distal ileum, and its systemic absorption is minimal [28, 29].

The feasibility of in vivo adeno-associated virus- (AAV-) mediated nonintegrating gene therapy has only been reported for PFIC type 3 clinically relevant mouse models. AAV-mediated gene therapy successfully prevented PFIC type 3 manifestation in a clinically relevant mouse model, representing a step forward in improving potential therapy options for PFIC type 3 patients [30]. In the case of PFIC type 1 and type 2, their pathophysiology is driven by individual cellular stressors. Therefore, all hepatocytes need to be corrected to stop damage-induced hepatocyte proliferation. The high vector dose to ensure transduction of all hepatocytes may cause liver toxicity. The therapeutic use of these procedures remains to be established even in mouse models.

Our study comprehensively reviewed the different clinical and treatment aspects of commonly encountered PFIC cases in Saudi Arabia. The study deals with a good number of PFIC type 3, which is not reported often in the literature. However, this paper also has limitations. We excluded patients missing data such as genetic confirmatory tests. We could not retrieve all the information of the patients enrolled as some of these patients were initially managed at different centers, including liver transplantation in some of them.

## 5. Conclusion

PFIC is considered one of the rare cholestatic liver diseases in Saudi Arabia with the most common type being PFIC type 3. The clinical presentation, laboratory tests, and outcome depend on the type of PFIC. The response to medical treatment was variable with an overall rate of liver transplantation of 64.6%. Following liver transplantation, the one-year survival rate was 96.1%, and the five-year survival rate was 96.1%. If patients are appropriately selected, liver transplantation offers an excellent survival benefit.

## Figures and Tables

**Table 1:** Baseline patients' characteristics.

	Overall (*N* = 79)
Gender	
Female	36 (45.6%)
Male	43 (54.4%)
Age at diagnosis of PFIC (months)	
Female mean (SD)	56.8 (53.1)
Male mean (SD)	68.3 (66.2)
Overall mean (SD)	63.1 (60.5)
Consanguinity between parents	
Missing data (*N*)	25
Yes	47 (87.0%)
Any other family members affected	
Missing data (*N*)	11
Yes	42 (61.8%)
Jaundice	39 (49.4%)
Pruritus	38 (48.1%)
Failure to thrive	8 (10.1%)
Bilirubin (*μ*mol/L)	
Mean (SD)	114.2 (152.2)
Range	3.0-811.0
ALT (U/L)	
Mean (SD)	189.0 (197.6)
Range	21.5-826.0
Alkaline phosphatase (U/L)	
Mean (SD)	531.2 (484.6)
Range	86.2-2577.0
GGT (IU/L)	
Mean (SD)	131.2 (146.7)
Range	8.0-691.0
Bile acid level (*μ*mol/L)	
Mean (SD)	204.4 (151.6)
Range	9.6-599.4
AFP (*μ*g/L)	
Mean (SD)	4268.8 (16324.5)
Range	0.6-100000.0
HCC	4 (5.1%)
Liver transplant rate (%)	51 (64.6%)
Duration of disease before liver transplant (months)	
Mean (SD)	53.9 (67.0)
Range	0.0-277.0
Death after liver transplant (%)	
Yes	2 (3.9%)
Recurrence after liver transplant (%)	
Yes	4 (7.8%)
Appearance of symptoms after liver transplant (months)	
Mean (SD)	22.5 (23.0)
Range	1.0-55.0
Survival after liver transplant (months)	
Mean (SD)	69.1 (44.1)
Range	0.0-199.0
Genetic alleles mutation	
Heterozygous	6 (16.2%)
Homozygous	31 (83.8%)

**Table 2:** Comparison between PFIC 2 and PFIC 3.

	PFIC 2	PFIC 3	Test statistic
	(*N* = 27)	(*N* = 47)	
Mean age at the time of data entry in years	8.63	13.2	*F*_1,72_ = 9.94, *P* < 0.013
Mean age at the time of diagnosis in months	22.1	86.4	*F*_1,71_ = 27.97, *P* < 0.013
Gender: male (%)	51.9	55.3	*Χ*21 = 0.08, *P* = 0.772
Family members affected (%)	52	70	*Χ*21 = 2.50, *P* = 0.112
Jaundice (%)	62.9	40.4	*Χ*21 = 3.49, *P* = 0.062
Pruritus (%)	40.7	53.2	*Χ*21 = 1.06, *P* = 0.302
Failure to thrive (%)	14.8	8.5	*Χ*21 = 0.71, *P* = 0.402
Coagulopathy (%)	37.01	40.43	*Χ*21 = 0.08, *P* = 0.772
Splenomegaly (%)	66.6	61.7	*Χ*21 = 0.18, *P* = 0.672
Hepatomegaly (%)	63	61.7	*Χ*21 = 0.01, *P* = 0.912
HCC (%)	7.4	4.25	*Χ*21 = 0.33, *P* = 0.562
Partial internal biliary diversion (%)	3.7	0.0	*Χ*21 = 1.87, *P* = 0.172
Liver transplantation (%)	70.37	57.45	*Χ*21 = 1.75, *P* = 0.192

^1^Kruskal-Wallis. ^2^Pearson. ^3^Wilcoxon.

**Table 3:** Laboratory results of PFIC 2 and PFIC 3.

Descriptives				
	PFIC types	Mean	Median	SD
Bilirubin (*μ*mol/L)	PFIC 2	137.1	108.4	116.7
	PFIC 3	63.13	20.35	90.2
ALT (U/L)	PFIC 2	308.33	289	259.8
	PFIC 3	113	87.15	81.6
Alkaline phosphatase (U/L)	PFIC 2	658.34	356	618.6
	PFIC 3	460.71	370.2	399.6
GGT (IU/L)	PFIC 2	35.15	31.5	23
	PFIC 3	193.35	145	161.1
Bile acid (*μ*mol/L)	PFIC 2	269.49	271	151.8
	PFIC 3	167.78	136	147
AFP (*μ*g/L)	PFIC 2	12575.11	22.6	26506
	PFIC 3	7.85	1.8	16.9

**Table 4:** Typical histological findings seen in PFIC types.

PFIC type	Type 1	Type 2	Type 3	Type 4
Histological features	Periportal and pericentrilobular fibrosis	Canalicular cholestasis, portal inflammation, and fibrosis with giant cells	Portal inflammation, portal fibrosis, cholestasis, ductal proliferation	Bland cholestasis, hepatic necrosis, ductal proliferation, portal fibrosis

**Table 5:** Patients who underwent liver transplantation.

	*N* = 51
Gender: male	28
PFIC types	
PFIC 1	3
PFIC 2	20
PFIC 3	27
PFIC 4	1
HCC: yes	4
Death after transplant	2
Recurrence after transplant	4
Persistent symptoms posttransplant	3
Symptoms control postliver transplant	47
Indication for liver transplantation	
Acute on chronic liver failure	1
Decompensated liver disease	36
HCC	4
Intractable pruritus	10

**Table 6:** Recurrence of the disease after LT in 4 patients with PFIC type 2.

	Clinical recurrence after liver transplantation (months)	Treatment provided	Response to treatment (yes/no)	Retransplantation
Patient 1	18	Plasmapheresis (5 cycles)	Yes	No
		IVIG (5 doses)		
		Rituximab (4 doses)		

Patient 2	1	Antithymocyte globulins (4 doses)	No	Listed for retransplantation
		Plasmapheresis (5 cycles)		
		Rituximab (5 cycles)		

Patient 3	16	Rituximab, IVIG, plasmapheresis	Yes	No

Patient 4	55	Plasmapheresis (5 cycles)	Yes	No
		IVIG (3 doses)		
		Rituximab (3 doses)		

## Data Availability

The data used to support the findings of this study are available from the corresponding author upon request. The corresponding author email is Alsohaibani@hotmail.com, and his phone number is +966114424729.

## Abbreviations

PFIC: Progressive familial intrahepatic cholestasis
ATP8B1: Adenosine triphosphatase, type 8B, member 1
ABCB11: Adenosine triphosphate-binding cassette, subfamily B, member 11
ABCB4: Adenosine triphosphate-binding cassette, subfamily B, member 4
REDCap: Research Electronic Data Capture
IRB: Institutional Review Board
RAC: Research Advisory Council
GGT: Gamma-glutamyl transferase
UDCA: Ursodeoxycholic acid
LT: Liver transplantation
HCC: Hepatocellular carcinoma
BSEB: Bile salt export pump
MDR3: Multidrug-resistant class III glycoprotein
TJP2: Tight junction protein 2
NR1H4: Nuclear receptor subfamily 1 group H member 4
MYO5B: Myosin 5B
FXR: Farnesoid X receptor
AAV: Adeno-associated virus.
